# Dr Erika Sutter 1917–2015

**Published:** 2015

**Figure:**
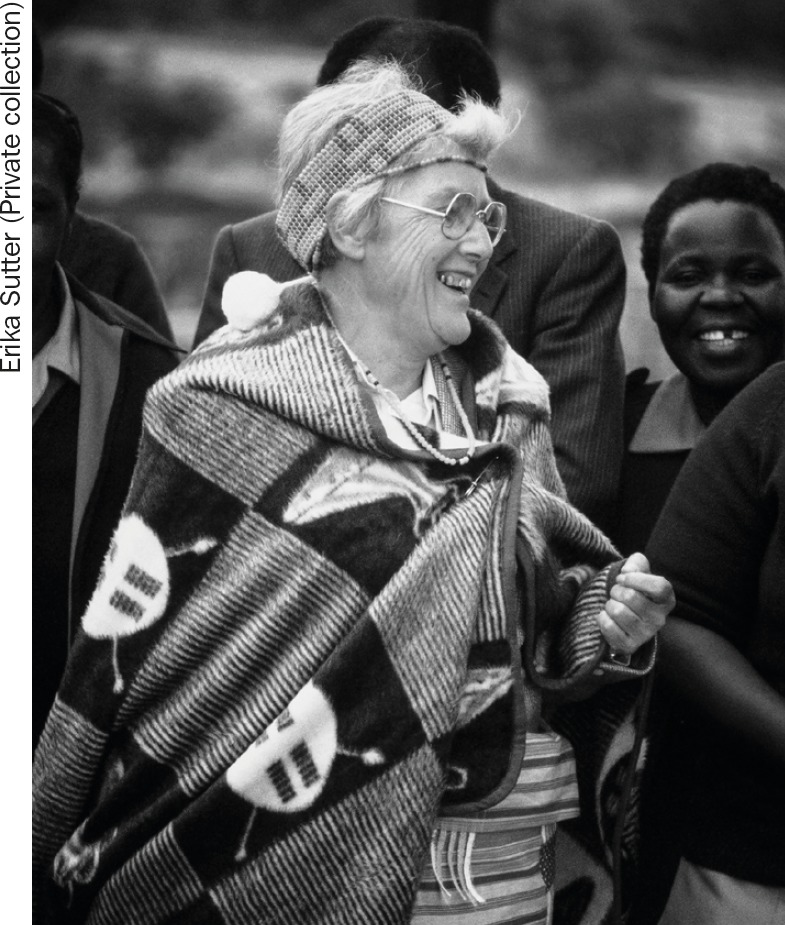


We put aside our sadness at the death of Erika Sutter, at the impressive age of 98, to celebrate the life of a truly remarkable pioneering ophthalmologist and inspirational human being.

Some of the *Community Eye Health Journal's* readers will have been touched by Erika's powerful promotion of community development in her capacity as an ophthalmologist, a teacher at the International Centre for Eye Health (ICEH) in London and the Swiss Tropical Institute in Basel, and author of academic papers and educational books, most notably *Hanyane, A Village Struggles for Eye Health.*

Erika Sutter was born and raised in Basel, Switzerland. After completing a doctorate in plant physiology in 1952, she worked for Roche and then the Swedish University of Agricultural Sciences. Throughout this time her faith guided her towards a missionary life. She joined the Swiss Mission in South Africa and took up the position of laboratory technician at the missionary hospital in Elim, an impoverished rural area in North-Eastern South Africa. Here her alertness to social disparities made her acutely aware of the injustices of apartheid and the need to address the political and social causes of disease.

In 1956, Erika was accepted to study medicine in South Africa. As there was a need for an eye specialist at Elim Hospital, she trained further in Switzerland, returning to South Africa in 1965 as a fully qualified ophthalmologist. She worked tirelessly at Elim Hospital while at the same time developing dreams fora comprehensive approach to blindness prevention. Her dreams were realised in three important projects: the introduction of a Diploma in Ophthalmic Nursing, the establishment of the Rivoni Rehabilitation Centre for Visually Impaired and Blind People, and, most notably, the Elim Care Group Project. Having treated the late effects of trachoma for many years, Erika realised that ‘it was necessary to come out of the hospital and to the people, there where the disease starts – in order to reach those who could still do something to prevent it.’ Working with Selina Maphorogo, she founded the Care Groups, village self-help groups working for better health in their communities. Within three years of starting the project, the prevalence of active trachoma had decreased by 50%. The movement is still strong more than 30 years later with around 2,000 members in over 200 villages working for improved health and social development.

Those of us who worked with Erika at ICEH admired her for her humility, her dogged determination to do a job properly with meticulous attention to detail, and her belief that the perspectives of the powerless-village women, and health workers low down in the medical hierarchy – are crucial to finding solutions.

Victoria Francis, London, August 2015
